# Bis(μ-*N*,*N*-dimethyl­dithio­carbamato-κ^3^ *S*,*S*′:*S*)bis­[(*N*,*N*-dimethyl­dithio­carbamato-κ^2^ *S*,*S*′)copper(II)]

**DOI:** 10.1107/S1600536809006230

**Published:** 2009-02-25

**Authors:** Le-Qing Fan, Ji-Huai Wu

**Affiliations:** a Institute of Materials Physical Chemistry and the Key Laboratory for Functional Materials of Fujian Higher Education, Huaqiao University, Quanzhou, Fujian 362021, People’s Republic of China

## Abstract

In the centrosymmetric dimeric title compound, [Cu_2_(C_3_H_6_NS_2_)_4_], the Cu^II^ atom is five-coordinate in a square-pyramidal environment. The basal coordination positions are occupied by four S atoms from two dimethyl­dithio­carbamate ligands and the apical coordination position is occupied by an S atom also bonded to the other Cu atom.

## Related literature

For the structural diversity and potential applications of transition metal complexes, see: Noro *et al.* (2000 ▶); Yaghi *et al.* (1998 ▶). For dialkyl­dithio­carbamates anions acting as monodentate, bidentate or bridging ligands, see: Engelhardt *et al.* (1988 ▶); Fernández *et al.* (2000 ▶); Koh *et al.* (2003 ▶). 
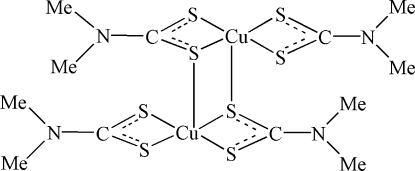


## Experimental

### 

#### Crystal data

[Cu_2_(C_3_H_6_NS_2_)_4_]
*M* *_r_* = 607.91
Monoclinic, 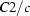

*a* = 8.068 (3) Å
*b* = 19.446 (7) Å
*c* = 15.108 (6) Å
β = 99.354 (6)°
*V* = 2338.7 (15) Å^3^
*Z* = 4
Mo *K*α radiation
μ = 2.54 mm^−1^
*T* = 293 K
0.25 × 0.20 × 0.15 mm

#### Data collection

Rigaku Mercury CCD diffractometer
Absorption correction: multi-scan (*CrystalClear*; Rigaku, 2007 ▶) *T*_min_ = 0.807, *T*_max_ = 1.000 (expected range = 0.552–0.683)
9796 measured reflections
2685 independent reflections
2423 reflections with *I* > 2σ(*I*)
*R*_int_ = 0.048

#### Refinement

*R*[*F* ^2^ > 2σ(*F* ^2^)] = 0.050
*wR*(*F* ^2^) = 0.141
*S* = 1.07
2685 reflections
118 parameters
H-atom parameters constrained
Δρ_max_ = 0.44 e Å^−3^
Δρ_min_ = −0.58 e Å^−3^

### 

Data collection: *CrystalClear* (Rigaku, 2007 ▶); cell refinement: *CrystalClear*; data reduction: *CrystalClear*; program(s) used to solve structure: *SHELXS97* (Sheldrick, 2008 ▶); program(s) used to refine structure: *SHELXL97* (Sheldrick, 2008 ▶); molecular graphics: *SHELXTL* (Sheldrick, 2008 ▶); software used to prepare material for publication: *SHELXTL*.

## Supplementary Material

Crystal structure: contains datablocks global, I. DOI: 10.1107/S1600536809006230/ng2548sup1.cif

Structure factors: contains datablocks I. DOI: 10.1107/S1600536809006230/ng2548Isup2.hkl

Additional supplementary materials:  crystallographic information; 3D view; checkCIF report

## Figures and Tables

**Table 1:** Selected bond lengths (Å)

Cu1—S3	2.3072 (13)
Cu1—S4	2.3208 (13)
Cu1—S1	2.3240 (13)
Cu1—S2	2.3278 (13)
Cu1—S1^i^	2.8258 (14)

Symmetry code: (i) 
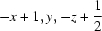
.

**Fig. 1.**
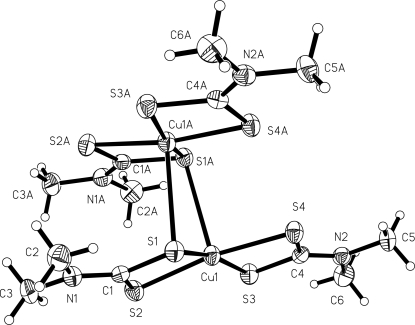
The molecular structure of (I) with 30% probability displacement ellipsoids (arbitrary spheres for H atoms). [Symmetry code: A 1 - x, y, 1/2 - z.]

## supplementary crystallographic information

### Comment

Research into transition metal complexes has been rapidly expanding because of their fascinating structural diversity, as well as their potential applications as functional materials and enzymes (Noro *et al.*, 2000; Yaghi *et al.*, 1998). Dialkyldithiocarbamates anions, which are typical sulfur ligands, acting as monodentate, bidentate or bridging ligands, are often chosen for the preparation of a considerable structural variety of complexes (Engelhardt *et al.*, 1988; Fernández *et al.*, 2000; Koh, *et al.*, 2003). We report here the crystal structure of the title copper(II) complex, (I), contanining a dimethyldithiocarbamate ligand.

The crystal structure of (I) is built up by dimeric entities of Cu^II^ complex (Fig. 1). The coordination geometry of Cu^II^ ion is described as a distorted square-pyramid. The basal coordination positions are occupied by four S atoms from two dimethyldithiocarbamate ligands. Each briding S atom simultaneously occupies an equatorial coordination site on one Cu^II^ ion and apical site on the other Cu^II^. The axial Cu—S bond distance is longer than the equatorial Cu—S ones (Table 1).

### Experimental

A mixture of Cu(Ac)_2_.H_2_O (0.04 g, 0.2 mmol) and NaS_2_CNMe_2_.2H_2_O (0.04 g, 0.2 mmol) was stirred in DMF (15 ml) at 313 K. 2-PrOH was diffused into the resulting solution, yielding single crystals of (I).

### Refinement

H atoms were positioned geometrically and refined as riding atoms, with C—H = 0.96 Å, *U*_iso_(H) = 1.5*U*_eq_(C).

### Crystal data

**Table:**

[Cu_2_(C_3_H_6_NS_2_)_4_]	*F*(000) = 1240
*M**_r_* = 607.91	*D*_x_ = 1.727 Mg m^−^^3^
Monoclinic, *C*2/*c*	Mo *K*α radiation, λ = 0.71073 Å
Hall symbol: -C 2yc	Cell parameters from 3071 reflections
*a* = 8.068 (3) Å	θ = 2.5–27.5°
*b* = 19.446 (7) Å	µ = 2.54 mm^−^^1^
*c* = 15.108 (6) Å	*T* = 293 K
β = 99.354 (6)°	Block, black
*V* = 2338.7 (15) Å^3^	0.25 × 0.20 × 0.15 mm
*Z* = 4	

### Data collection

**Table:**

Rigaku Mercury CCD diffractometer	2685 independent reflections
Radiation source: Sealed Tube	2423 reflections with *I* > 2σ(*I*)
Graphite Monochromator	*R*_int_ = 0.048
ω scans	θ_max_ = 27.5°, θ_min_ = 2.1°
Absorption correction: multi-scan (*CrystalClear*; Rigaku, 2007)	*h* = −9→10
*T*_min_ = 0.807, *T*_max_ = 1.000	*k* = −25→25
9796 measured reflections	*l* = −19→18

### Refinement

**Table:**

Refinement on *F*^2^	Primary atom site location: structure-invariant direct methods
Least-squares matrix: full	Secondary atom site location: difference Fourier map
*R*[*F*^2^ > 2σ(*F*^2^)] = 0.050	Hydrogen site location: inferred from neighbouring sites
*wR*(*F*^2^) = 0.141	H-atom parameters constrained
*S* = 1.07	*w* = 1/[σ^2^(*F*_o_^2^) + (0.0741*P*)^2^ + 4.6176*P*] where *P* = (*F*_o_^2^ + 2*F*_c_^2^)/3
2685 reflections	(Δ/σ)_max_ < 0.001
118 parameters	Δρ_max_ = 0.44 e Å^−^^3^
0 restraints	Δρ_min_ = −0.58 e Å^−^^3^

### Special details

**Table:**

Geometry. All e.s.d.'s (except the e.s.d. in the dihedral angle between two l.s. planes) are estimated using the full covariance matrix. The cell e.s.d.'s are taken into account individually in the estimation of e.s.d.'s in distances, angles and torsion angles; correlations between e.s.d.'s in cell parameters are only used when they are defined by crystal symmetry. An approximate (isotropic) treatment of cell e.s.d.'s is used for estimating e.s.d.'s involving l.s. planes.
Refinement. Refinement of *F*^2^ against ALL reflections. The weighted *R*-factor *wR* and goodness of fit *S* are based on *F*^2^, conventional *R*-factors *R* are based on *F*, with *F* set to zero for negative *F*^2^. The threshold expression of *F*^2^ > σ(*F*^2^) is used only for calculating *R*-factors(gt) *etc*. and is not relevant to the choice of reflections for refinement. *R*-factors based on *F*^2^ are statistically about twice as large as those based on *F*, and *R*- factors based on ALL data will be even larger.

### Fractional atomic coordinates and isotropic or equivalent isotropic displacement parameters (Å^2^)

**Table:**

	*x*	*y*	*z*	*U*_iso_*/*U*_eq_	
Cu1	0.58962 (6)	0.37634 (2)	0.36682 (3)	0.04294 (19)	
S1	0.72601 (13)	0.39120 (5)	0.24400 (7)	0.0438 (3)	
S2	0.66137 (15)	0.49238 (5)	0.37410 (7)	0.0493 (3)	
S3	0.50794 (15)	0.35823 (5)	0.50440 (7)	0.0497 (3)	
S4	0.56076 (14)	0.25771 (5)	0.37160 (7)	0.0478 (3)	
N1	0.7709 (4)	0.52631 (17)	0.2215 (2)	0.0482 (8)	
N2	0.4789 (4)	0.22341 (17)	0.5308 (2)	0.0451 (7)	
C1	0.7268 (5)	0.47699 (19)	0.2734 (3)	0.0410 (8)	
C2	0.8173 (7)	0.5106 (3)	0.1342 (3)	0.0660 (13)	
H2A	0.8225	0.4616	0.1269	0.099*	
H2B	0.9251	0.5303	0.1307	0.099*	
H2C	0.7348	0.5295	0.0876	0.099*	
C3	0.7562 (7)	0.5986 (2)	0.2445 (4)	0.0642 (13)	
H3A	0.7251	0.6022	0.3030	0.096*	
H3B	0.6717	0.6201	0.2011	0.096*	
H3C	0.8620	0.6211	0.2444	0.096*	
C4	0.5123 (5)	0.27279 (19)	0.4761 (2)	0.0396 (8)	
C5	0.4841 (6)	0.1509 (2)	0.5072 (3)	0.0616 (12)	
H5A	0.5085	0.1466	0.4473	0.092*	
H5B	0.3773	0.1301	0.5103	0.092*	
H5C	0.5699	0.1281	0.5483	0.092*	
C6	0.4417 (6)	0.2385 (3)	0.6202 (3)	0.0631 (13)	
H6A	0.4415	0.2874	0.6291	0.095*	
H6B	0.5257	0.2178	0.6645	0.095*	
H6C	0.3333	0.2202	0.6258	0.095*	

### Atomic displacement parameters (Å^2^)

**Table:**

	*U*^11^	*U*^22^	*U*^33^	*U*^12^	*U*^13^	*U*^23^
Cu1	0.0524 (3)	0.0380 (3)	0.0398 (3)	0.00016 (19)	0.0115 (2)	0.00059 (18)
S1	0.0478 (6)	0.0412 (5)	0.0442 (5)	0.0025 (4)	0.0125 (4)	−0.0010 (4)
S2	0.0613 (7)	0.0414 (5)	0.0462 (6)	−0.0044 (4)	0.0114 (5)	−0.0078 (4)
S3	0.0667 (7)	0.0447 (5)	0.0397 (5)	0.0014 (5)	0.0144 (5)	−0.0027 (4)
S4	0.0643 (7)	0.0385 (5)	0.0430 (6)	0.0041 (4)	0.0158 (5)	−0.0005 (4)
N1	0.0478 (19)	0.0466 (18)	0.0489 (19)	−0.0053 (15)	0.0041 (15)	0.0071 (15)
N2	0.0445 (18)	0.0467 (17)	0.0438 (18)	−0.0017 (14)	0.0063 (14)	0.0063 (14)
C1	0.0365 (18)	0.0425 (18)	0.041 (2)	−0.0001 (15)	−0.0015 (15)	0.0027 (15)
C2	0.069 (3)	0.072 (3)	0.059 (3)	−0.008 (2)	0.018 (2)	0.013 (2)
C3	0.076 (3)	0.042 (2)	0.071 (3)	−0.012 (2)	0.002 (2)	0.008 (2)
C4	0.0359 (18)	0.0454 (19)	0.0367 (18)	0.0026 (15)	0.0034 (14)	0.0051 (15)
C5	0.071 (3)	0.043 (2)	0.070 (3)	−0.002 (2)	0.009 (2)	0.013 (2)
C6	0.071 (3)	0.072 (3)	0.050 (3)	−0.005 (2)	0.021 (2)	0.016 (2)

### Geometric parameters (Å, °)

**Table:**

Cu1—S3	2.3072 (13)	N2—C5	1.457 (5)
Cu1—S4	2.3208 (13)	C2—H2A	0.9600
Cu1—S1	2.3240 (13)	C2—H2B	0.9600
Cu1—S2	2.3278 (13)	C2—H2C	0.9600
Cu1—S1^i^	2.8258 (14)	C3—H3A	0.9600
S1—C1	1.726 (4)	C3—H3B	0.9600
S2—C1	1.715 (4)	C3—H3C	0.9600
S3—C4	1.717 (4)	C5—H5A	0.9600
S4—C4	1.713 (4)	C5—H5B	0.9600
N1—C1	1.324 (5)	C5—H5C	0.9600
N1—C2	1.461 (6)	C6—H6A	0.9600
N1—C3	1.457 (6)	C6—H6B	0.9600
N2—C4	1.323 (5)	C6—H6C	0.9600
N2—C6	1.460 (5)		
			
S3—Cu1—S4	77.03 (4)	H2A—C2—H2B	109.5
S3—Cu1—S1	168.48 (5)	N1—C2—H2C	109.5
S4—Cu1—S1	102.20 (4)	H2A—C2—H2C	109.5
S3—Cu1—S2	102.16 (4)	H2B—C2—H2C	109.5
S4—Cu1—S2	170.94 (5)	N1—C3—H3A	109.5
S1—Cu1—S2	76.75 (4)	N1—C3—H3B	109.5
S3—Cu1—S1^i^	100.81 (5)	H3A—C3—H3B	109.5
S4—Cu1—S1^i^	91.99 (4)	N1—C3—H3C	109.5
S1—Cu1—S1^i^	90.70 (4)	H3A—C3—H3C	109.5
S2—Cu1—S1^i^	97.01 (4)	H3B—C3—H3C	109.5
C1—S1—Cu1	84.09 (14)	N2—C4—S3	122.2 (3)
C1—S2—Cu1	84.21 (13)	N2—C4—S4	123.5 (3)
C4—S3—Cu1	84.45 (13)	S3—C4—S4	114.3 (2)
C4—S4—Cu1	84.12 (13)	N2—C5—H5A	109.5
C1—N1—C2	121.1 (4)	N2—C5—H5B	109.5
C1—N1—C3	121.2 (4)	H5A—C5—H5B	109.5
C2—N1—C3	117.3 (4)	N2—C5—H5C	109.5
C4—N2—C6	121.7 (4)	H5A—C5—H5C	109.5
C4—N2—C5	122.2 (4)	H5B—C5—H5C	109.5
C6—N2—C5	116.1 (4)	N2—C6—H6A	109.5
N1—C1—S2	123.4 (3)	N2—C6—H6B	109.5
N1—C1—S1	122.5 (3)	H6A—C6—H6B	109.5
S2—C1—S1	114.1 (2)	N2—C6—H6C	109.5
N1—C2—H2A	109.5	H6A—C6—H6C	109.5
N1—C2—H2B	109.5	H6B—C6—H6C	109.5

Symmetry codes: (i) −*x*+1, *y*, −*z*+1/2.
